# Social connectedness and disconnectedness in individuals with recent onset psychosis and suicidal experiences: a systematic review of the evidence

**DOI:** 10.1186/s12888-026-08092-z

**Published:** 2026-05-13

**Authors:** Kamelia Harris, Gillian Haddock, Sarah Peters, Lee D. Mulligan, Patricia Gooding

**Affiliations:** 1 Division of Psychology and Mental Health, School of Health Sciences, Faculty of Biology, Medicine and Health, University of Manchester, Manchester, UK; 2 Manchester Academic Health Sciences Centre (MAHSC), University of Manchester, Manchester, UK; 3 Greater Manchester Mental Health NHS Foundation Trust (GMMH), Manchester, UK; 4 Manchester Centre for Health Psychology, Division of Psychology and Mental Health, School of Health Sciences, Faculty of Biology, Medicine and Health, University of Manchester, Manchester, UK

**Keywords:** Social connectedness, Social disconnectedness, Recent onset psychosis, Suicidality, Suicidal experiences, Systematic literature review

## Abstract

**Background:**

Suicidal experiences (e.g. suicidal thoughts, plans, urges, compulsions, images, acts, attempts) are common in the early stages of psychosis and represent a global healthcare concern. As well as hallucinations and delusions, psychosis is associated with difficulties in forming interpersonal relationships, causing isolation, disconnectedness, and significant psychological distress. This systematic literature review aimed to examine the effects of perceptions of offline and online social connectedness and disconnectedness on suicidal experiences in people with recent onset psychosis. We proposed a Social Connectedness and Disconnectedness (SoCaD) conceptual framework comprising six domains which guided the analytic process.

**Methods:**

A convergent, sequential explanatory approach to analysis was used. Fourteen studies were included from four electronic databases (i.e. PsycInfo, Embase, MEDLINE, Web of Science). The study screening and quality assessment procedures were checked by an independent researcher.

**Results:**

Findings pertaining to five SoCaD domains were identified: 1. Supportive relationships with others; 2. Social identity and purpose; 3. A sense of belonging; 4. Perceived social value; and 5. A sense of mattering to others. No studies were identified that specifically examined experiences and perceptions of social connectedness or disconnectedness in the context of online social media activity and communication. This represents a substantial gap in the evidence.

**Discussion:**

Overall, only a few studies made a useful contribution to better understanding the relationships between social connectedness and disconnectedness, recent onset psychosis, and suicidal experiences. Future research should methodically examine domains of social connectedness and disconnectedness across offline and online contexts and focus on context-specific understanding of these social dynamics to enhance suicide prevention strategies in this vulnerable population.

**Clinical trial number:**

Not applicable.

**Supplementary information:**

The online version contains supplementary material available at 10.1186/s12888-026-08092-z.

## Background

Psychosis is a severe mental health problem characterised by experiences such as hearing voices, paranoia, and feelings of persecution [1]. These experiences can be extremely frightening, distressing, and are often accompanied by suicidal thoughts, behaviours, attempts, and suicide death [2]. People with psychosis are approximately five times more likely to experience suicidal thoughts and ten times more likely to attempt suicide compared to people without psychosis [3]. The lifetime suicide risk in people with psychosis has been documented as between 2% and 13% [4–7] but this is particularly heightened in the early stages of psychosis (i.e., the prodromal period; people at clinically high risk of psychosis; people with recent onset psychosis, e.g., those accessing UK early intervention services) which has been associated with a 60% increased suicide risk, compared to the later stages (i.e., people with a formal schizophrenia diagnosis [8, 9]). Therefore, it is particularly vital to understand the precursors of, and counters to, suicidal experiences in people with recent onset psychosis.

Psychosis typically develops in adolescence or young adulthood when individuals are learning how to form social relationships [10, 11]. This period can make the early stages of psychosis especially challenging, as it overlaps with changes in peer relationships and social responsibilities [10]. Both psychosis and suicidal experiences (including thoughts, plans, urges, compulsions, images, acts, attempts) can make individuals feel un-understandable, to self-isolate, not trust others, and experience stigma from self and others [12–18]. For these reasons, people with recent onset psychosis tend to have diminished or fragmented social networks, reduced contact with others, and difficulties in maintaining interpersonal relationships [11, 12, 19–21]. These experiences have often been framed as having a lack of social connections or social disconnectedness [22, 23].

Social connectedness has been conceptualised as perceptions of belonging to a social group, community, relationship, or network, and engaging with and maintaining relationships within different social networks and support systems [24–26]. High levels of social connectedness have been proposed to be important determinants for positive mental health [24, 25, 27–30]. Specifically, a greater sense of social connectedness has been associated with reduced suicidal thoughts, attempts, and deaths in adolescent and adult clinical and non-clinical samples [31–34]. Perceptions of social connectedness are not merely considered to be the opposite of disconnectedness but represent differentiable dimensions. For example, an individual may experience a sense of belonging to family, yet also feel excluded, rejected and/or profoundly disconnected from communities and society more generally because of stigma and lack of understanding [35–37]. Relatedly, being surrounded by others may not contribute to an increased sense of connectedness or prevent feelings of loneliness or isolation, because even when others’ intentions are to be supportive, they may not be perceived as such nor experienced as positive [38–40]. In contrast, social disconnectedness often includes perceptions of loneliness, isolation, not belonging, invisibility, insignificance, being passively or actively excluded by others, and a lack of closeness or intimacy within romantic or sexual relationships [24, 28, 41–46].

A recent global development of potential transformative importance to social connectedness is the use of the Internet and social media platforms [47]. Whilst social media platforms have a wide appeal across the lifespan, they are particularly attractive to younger people [48, 49], including those experiencing recent onset psychosis [50–52]. Such individuals frequently use online social media platforms to socialise, form friendships, share information, feel part of communities, learn about mental health, and navigate the challenges related to psychosis [53]. Despite these advantages, online social media use also poses risks, such as the spread of misinformation about mental health problems, harassment, cyberstalking and cyberbullying [54], with consequent exacerbation of poor mental health, including suicidal experiences [55, 56]. It is clearly crucial to understand the extent to which online social interactions facilitate the formation of supportive, empowering, and inclusive social connections or, conversely, diminish those connections and how this may influence psychosis and suicidal experiences [50, 52].

Hence, there are three gaps in the current literature. The first is the need for a multi-dimensional approach to investigating social connectedness and disconnectedness that goes beyond isolation and lack of support, to include feelings of insignificance, invisibility, lack of belongingness [57, 58], and digital age alienation [59]. The second is the need to understand how using social media to form networks and relationships may precipitate or amplify suicidal experiences in young people experiencing recent onset psychosis. The third is a lack of understanding of how specific mental health problems, such as psychosis, interact with perceptions of social connectedness and disconnectedness in pathways to suicidality, particularly in young people who are forming identities and navigating life transitions [52, 60] and are vulnerable to developing psychosis and concomitant suicidal experiences. The aim of this literature review was to address these three gaps by systematically reviewing the extant evidence. In relation to this aim, the following research question was addressed: *In what ways do perceptions of social connectedness and social disconnectedness affect suicidal experiences in people with recent onset psychosis?*

**Fig. 1:**
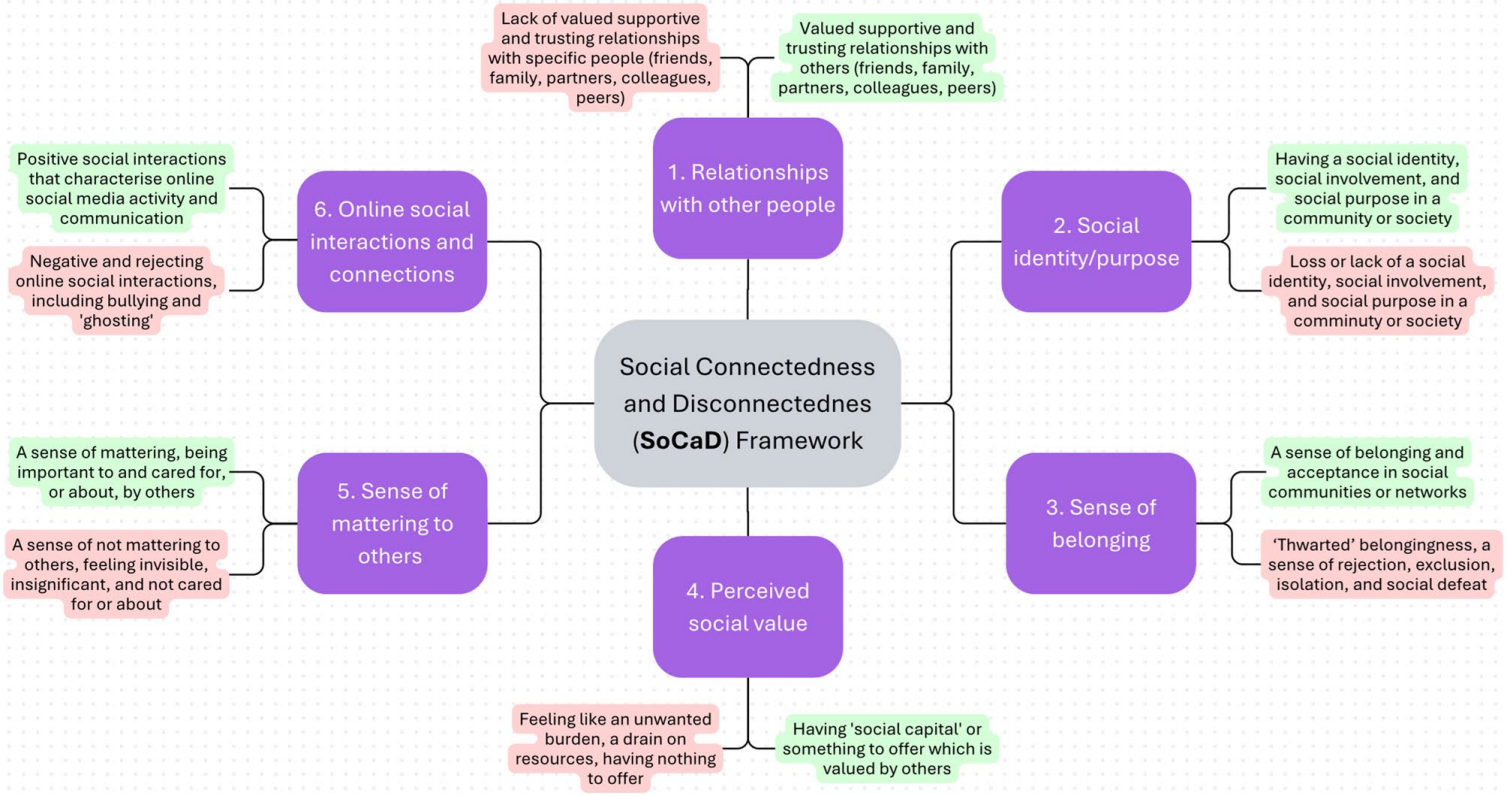
The social connectedness and disconnectedness (SoCaD) framework as applied to suicidal experiences

To address this research question, it was important to ground it within a multi-componential framework in order to systematically examine the different domains of social connectedness and disconnectedness. It is notable that, although contemporary models of suicide posit, as central to suicidal experiences, appraisals of i. a lack of social support and connectedness (Schematic Appraisals Model of Suicide (SAMS) [61]); ii. burdensomeness and thwarted belongingness (Interpersonal theory of Suicide (IPTS) [62]); and iii. a loss or lack of social connections, social integration, and social purpose (Three-Step Model of suicide (3ST [63]), none have described in detail the different kinds of disconnectedness that may be operating in pathways to suicide. A recent systematic literature review identified a conceptual social connectedness framework comprising i. Closeness, ii. Identity and common bonds, iii. Valued relationships, iv. Involvement, and being v. Cared for and accepted (CIVIC [64]). Furthermore, WHO [26] described three dimensions of social connectedness, namely, i. structure (i.e., number/types of social relationships), ii. function (i.e., type and amount of support), and iii. quality (i.e., appraisals of the quality of social relationships, e.g., positive, satisfying, negative, conflictual, strained). Neither the contemporary models of suicide, nor the CIVIC conceptual framework or the WHO description of social connectedness feature elements pertaining to the impact of online interpersonal relationships/networks on mental health and suicidal experiences. Consequently, we amalgamated the elements highlighted by CIVIC and WHO with other relevant literature and models of suicide to form the Social Connectedness and Disconnectedness (SoCaD) framework (see Fig. 1 for the framework and Table 1 describing the sources that underpinned the six domains of the framework). We organised, evaluated, and synthesised the literature relevant to the research question using the SoCaD framework.

**Table 1:** Development of the six domains comprising the social connectedness and disconnectedness (SoCaD) framework based on evidence from existing literature and conceptual frameworks/models

SoCaD domain	Conceptual framework/model	Evidence from the literature*
1. Valued supportive relationships with other people	Closeness, identity and common bond, and valued relationships elements (CIVIC framework) [64], Social support and connectedness (Schematic Appraisals Model of Suicide [SAMS] [61], Social connections (Three-Step Theory of Suicide [3ST] [63],	Baumeister RF, Leary MR. The need to belong: desire for interpersonal attachments as a fundamental human motivation. 1995 [24] World Health Organization definition of social connection [26] Gooding et al. The interplay between suicidal experiences, psychotic experiences and interpersonal relationships. 2023 [57] Harris K et al. Psychological resilience to suicidal thoughts and behaviours in people with schizophrenia. 2019
2. Having a social identity and purpose	Identity and common bond element (CIVIC framework) [64], Social purpose (Three-Step Theory of Suicide [3ST]) [63],	Klonsky ED, May AM. The Three-Step Theory (3ST): a new theory of suicide rooted in the ideation-to-action framework. 2015 [63] Flett G. Interpersonal stress, mattering. 2018; 2021 [41, 42] Harris K et al. Psychological resilience to suicidal thoughts and behaviours in people with schizophrenia. 2019
3. Having a sense of belonging	Closeness, and cared for and accepted elements (CIVIC framework) [64], Socials integration (Three-Step Theory of Suicide [3ST]) [63], Burdensomeness, thwarted belongingness (Interpersonal-Psychological Theory of Suicide [IPTS]) [58],	Van Orden KA et al. The interpersonal theory of suicide. 2010 [62] Baumeister RF, Leary MR. The need to belong: desire for interpersonal attachments as a fundamental human motivation. 1995 [24] World Health Organization definition of social connection [26]
4. Perceived social value	Involvement element (CIVIC framework) [64], Burdensomeness (Interpersonal-Psychological Theory of Suicide [IPTS]) [58],	Joiner TE. *Why people die by suicide*. 2005 [58] Van Orden KA et al. The interpersonal theory of suicide. 2010 [62] Lin N. Social capital: a theory of social structure and action. 2002
5. A sense of mattering to others	Cared for and accepted element (CIVIC framework) [64],	Flett G. Interpersonal stress, mattering. 2018; 2021 [41, 42] World Health Organization definition of social connection [26] Gooding et al. The interplay between suicidal experiences, psychotic experiences and interpersonal relationships. 2023 [57];
6. Online social interactions and connections		Bjornestad J et al. Social media and social functioning in psychosis: a systematic review. 2019 [50] Highton-Williamson E. et al. Online social networking in people with psychosis: a systematic review. 2015 [53] Memon AM et al. Online social networking and suicidality in adolescents. 2018 [56]

*Note: The framework was also informed by authors’ work with people with lived experience of psychosis and suicidal thoughts, plans, acts, urges, and behaviours

## Methods

### Design, data analysis and synthesis strategy

We conducted this review in accord with the PRISMA guidelines for systematic literature reviews [65]. The protocol was registered on PROSPERO (ref: CRD420251107590). The protocol did not include the implementation of the SoCaD framework and it was not initially a part of the data analysis plan. The framework was developed iteratively and in parallel with the analytic process, and subsequently informed and extended the data analysis and synthesis. This did not result in changes to study eligibility decisions or alteration of findings but provided an analytic scaffold for examining data in greater conceptual depth.

A results-based convergent, sequential explanatory design was used in which findings from the quantitative and qualitative studies were analysed separately using different synthesis methods and results of both syntheses were then integrated [66, 67]. Specifically, quantitative studies were first collated and summarised using narrative synthesis focusing on reported between-participant/group differences or relationships between recent onset psychosis, social connectedness/disconnectedness, and suicidal experiences [68]. Findings from the included qualitative studies were collated in accord with the principles of meta-ethnography to explain and expand the findings identified across the quantitative studies [69, 70].

The synthesis of qualitative studies employed Noblit and Hare’s [69] seven-steps of meta-ethnography, comprising: 1. getting started, 2. deciding what is relevant, 3. reading the studies, 4. determining how the studies are related, 5. translating the studies into one another, 6. synthesising translations, and 7. expressing the synthesis. To enable synthesis and convergence, we focused on collating findings across qualitative and quantitative studies which were broadly comparable (i.e., replicative), refutational (i.e., in opposition to each other) and facilitated the development of a novel conceptual understanding or mechanism [71, 72].

Results which were relevant to the research question were categorised according to the domains of the proposed SoCaD framework (see Fig. 1). The integration consisted of iterative comparisons of quantitative and qualitative findings. The following questions guided this process [73]:

1. To what extent is the evidence from the quantitative and qualitative studies supportive or contradictory?
2. To what extent does the quantitative and qualitative evidence explain/elaborate the relationships between online/offline social connectedness and disconnectedness, recent onset psychosis, and suicidal experiences?
3. To what extent does the qualitative evidence explain differences in these relationships across the quantitative studies?
4. To what extent do the qualitative studies include or omit aspects highlighted in the quantitative evidence?

The analysis was grounded in a pragmatic epistemology which is a flexible paradigm for integrating data from diverse research methods and perspectives [74–76].

### Search strategy

Four electronic databases, namely, PsycInfo, Embase, MEDLINE, and Web of Science, were searched for eligible studies published between the first indexed year of the databases and June 2025. Results were limited to full-text articles which were peer-reviewed, published in English, and included human participants. The searches were re-run in December 2025 and no new, eligible papers were identified for inclusion in the review.

Definitions of key concepts and search strategies used in the extant literature (e.g. [13, 30, 50, 53]), and discussions with academics, a clinician, and people with lived experience of recent onset psychosis and suicidal experiences all contributed to the development of the search strategy for this review (see Supplementary Table S1 for the search terms). The search strategy was guided by the target population and reflected the six SoCaD framework domains (Fig. 1). Specifically, it incorporated terms relating to experiences of recent onset psychosis (e.g., At-Risk Mental States [ARMS], Ultra High Risk [UHR], Clinical High Risk [CHR], First Episode Psychosis [FEP]), terms relating to the domains in the social connectedness and disconnectedness framework, and terms relating to suicidal experiences (i.e., suicidal thoughts, plans, acts, urges, compulsions, images, acts, attempts, behaviours). Of note, the search strategy incorporated a ‘NOT’ Boolean operator with terms which excluded studies on irrelevant topics, such as pharmacology, neuroscience, brain imaging, perinatal literature, non-suicidal self-injury, assisted suicide.

### Study eligibility criteria

The were five inclusion criteria:

i. Studies conducted in any country and including participants who were aged 14 years or older (based on the lower age cut-off that is typically used in UK early intervention mental health services) or with a mean sample age of 14 years or over.
ii. Studies providing indicant(s) of recent onset psychosis experiences, suicidal experiences, and social connectedness and/or disconnectedness, for example:
  - Participants were described as currently being at risk of psychosis (e.g., ultra-high risk [UHR], clinically high risk [CHR], at-risk mental state [ARMS]), experiencing first episode psychosis (FEP), or broadly described as experiencing recent onset non-affective psychosis, or being in the early stages of psychosis. The nature and severity of these experiences could be a) self-reported, b) determined by a clinician/trained researcher, c) based on information from clinical/medical notes, or d) based on reported psychiatric diagnostic categories using manuals, such as the Diagnostic and Statistical Manual of Mental Disorders (DSM [77]) or the International Statistical Classification of Diseases and Related Health Problems (ICD-10 [78]).
  - Participants were described as having any type of suicidal experience, including thoughts, intentions, plans, urges, compulsions, images, acts, and attempts at any point in their life.
  - Participants reported current perceptions/feelings of social connectedness and/or disconnectedness, experienced in the context of in-person and/or online interactions with people and/or groups or communities of people.
iii. Data had to comprise participants’ own accounts, perceptions, and experiences of recent onset psychosis, social connectedness and/or disconnectedness, and suicidal experiences.
iv. Quantitative, qualitative, convergent, mixed methods studies, case reports and case studies.
  - Any cross-sectional, longitudinal or micro-longitudinal quantitative designs (e.g., trials, experimental, quasi-experimental, survey, descriptive) including those testing within and/or between-participant comparisons, associations, models of relationships (e.g., moderation, mediation, moderated mediation), and models of pathways (e.g., network models, Structural Equation Models, Profile Analyses).
  - Any qualitative designs (e.g., ethnography, grounded theory, phenomenology, narrative, case study) using different data elicitation methods (e.g., interviews, images, photographs, art), conducted at one time point or over time.
v. Empirical studies published in English in peer-reviewed academic journals.

The following exclusion criteria were applied:

i. Studies reporting perceptions of connectedness or disconnectedness that were not borne of being with other people. For example, connectedness with animals, pets, nature, value systems (e.g., human rights), beliefs (e.g., communism), objects, places, abstract concepts (e.g., justice), religions, religious deities, and the spiritual world.
ii. Studies reporting only structural or demographic indicants of connectedness or disconnectedness, for example, the size of social networks (e.g., number of friends), living situation (e.g., living alone, with others), or relationship status (e.g., being single, married, in a relationship).
iii. Studies reporting on general occupational or social functioning and adjustment (e.g., in work, school, or family settings and environments) and the extent to which an individual felt that they were abiding by or conforming to social norms, rules, and guidelines.
iv. Studies which did not report the scores on sub-scales or items of measures that capture perceptions of social connectedness or disconnectedness as described in the SoCaD framework. If relevant data could not be extracted (e.g., the study provided a total score on a resilience measure, for example, rather than a relevant sub-scale score about resilience related to social support), the study was excluded.
v. Studies reporting carers’, professionals’, significant others’, family members’ or friends’ perceptions of recent onset psychosis, social connectedness and/or disconnectedness, and suicidal experiences in participants.
vi. Books, book chapters, handbooks, literature reviews, meta-analyses, meta-syntheses, theses, dissertations, abstracts, conference proceedings and abstracts, guidelines, study protocols, commentaries, position papers, corrigenda, letters to editors, and studies from the grey literature.

### Study screening

End Note was used to collate and manage the articles extracted from the four databases. The study screening was conducted by the first author (KH) and included four stages:

i. Removing duplicate articles.
ii. Title and abstract screening. If the study was missing an abstract, it was included at this stage and reviewed in the full-text screening stage.
iii. Full-text screening of potentially eligible articles.
iv. Screening the reference lists of included papers for potentially eligible articles that were not identified in the electronic database searches (i.e., backward citation tracing), and forward citation tracing of all articles that cited the included papers.

An independent researcher (LM) checked 10% of the results against the eligibility criteria to ascertain reliability at title and abstract and full-text screening stages. Both Cohen’s kappa (*κ*) and the percent agreement were calculated and used to indicate the level of agreement between the first author, KH and LM [79, 80]. Kappa values between .01 and .20 indicate slight agreement, between .21 and .40 fair, between .41 and .60 moderate, between .61 and .80 substantial, and over .81 perfect agreement [79]. A percent agreement value of 80% is the minimum acceptable agreement between independent raters [80].

### Data extraction

Following the study screening stage, information relevant to the research question from each included study was extracted by the first author (KH) into an Excel spreadsheet with the following headings: study authors, publication year, research design, research aim(s), sample size, sampling strategy, sample characteristics (e.g., age, gender, diagnosis), psychosis measures, social connectedness/disconnectedness measures, type of social connectedness and disconnectedness (as described in the SoCaD framework shown in Fig. 1), suicide measures, analysis approach, and results. The relevant information extracted into the Excel spreadsheet from all included studies was rigorously checked against the published papers by the fourth author (LM). One discrepancy was identified in relation to an incorrectly recorded study sample size (*κ* = 0.98; 99% agreement).

### Quality assessment

The Mixed Methods Appraisal Tool (MMAT [81]); was used for assessing the quality of the included studies. The tool consists of five methodological design study categories: 1. qualitative (including case studies), 2. quantitative randomised controlled trials, 3. quantitative non-randomised, 4. quantitative descriptive, and 5. mixed methods studies. Each study category comprises five items appraising the methodological quality of the included studies.

In addition, one item from the Critical Appraisal Skills Programme (CASP) Qualitative Checklist [82], which assessed reflexivity, i.e., the relationship between the researcher, the research processes, and collected data, was incorporated into the qualitative study category of the MMAT. Specifically, this item assessed whether the researcher critically examined their own biases and influence on the research process and whether they responded to or considered the implications of any events during the study (e.g., power imbalance in interviews). Reflexivity is a fundamental part of qualitative research which considers the researcher’s perspective, awareness of, and influence on the research processes, such as study design, data collection, and interpretation [83]. A failure to incorporate reflexivity can negatively impact the analytical process and the interpretations and representations of participant experiences [84].

The total number of items for quantitative studies in the MMAT was five, and the total number of items for qualitative studies was six (see Supplementary Table S2 for the quality assessment tool). There were three response categories for each item, namely, “yes”, “no” and “can’t tell”. The overall quality rating included five levels: low, low-moderate, moderate, moderate-high, and high. The rating was determined by the number of “yes” responses to the items of the quality assessment tool. For example, if a qualitative study had four “yes” responses out of six items, its overall quality would be moderate-high. Similarly, if a quantitative study had two “yes” responses out of five items, the quality rating would be low-moderate. Two screening items were applied to all study categories. These two screening items checked if there were clear research questions/aims and whether the collected data allowed the research questions/aims to be addressed. Quality appraisal was not feasible if the answer was “no” or “can’t tell” to one or both screening questions (see Supplementary Table S2 for the quality assessment tool). The first author (KH) and an independent researcher (LM) rated the quality of all included studies using the quality assessment tool. Discrepancies were discussed between the two raters and resolved.

## Results

### Overview of the included studies

**Fig. 2:**
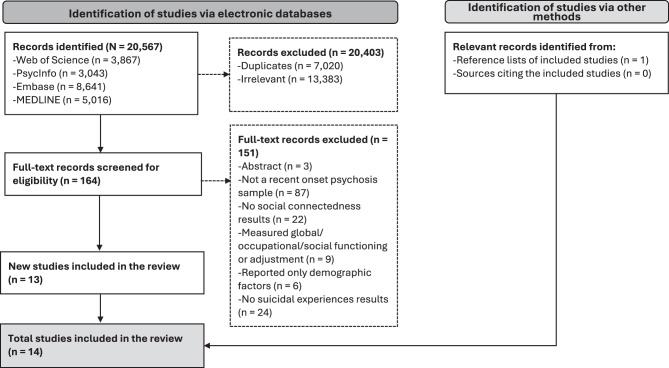
PRISMA flowchart

A total of 20,567 articles were found across the four databases. Of these, 13 were eligible for inclusion in the review and one additional relevant article was identified through backward citation tracing, resulting in a total of 14 studies (see Fig. 2 for the PRISMA flowchart).

Cohen’s kappa showed a moderate inter-rater agreement at the title and abstract screening stage (*κ* = .47) and perfect agreement according to the percent agreement approach (99%). Similarly, at the full-text screening stage Cohen’s kappa showed moderate inter-rater agreement (*κ* = .48) and acceptable agreement when using the percent agreement approach (81%). The inconsistency in inter-rater reliability is known as the ‘kappa paradox’ [85], whereby there is a strong inter-rater percent agreement, but the kappa value is lower. This often leads to the incorrect conclusion that inter-rater agreement is lacking. As a result, relying on the kappa statistic can distort the evaluation and may lead to biased assessment outcomes. Therefore, we have reported both Cohen’s kappa and percent agreement values.

The were two discrepancies between the two raters at the full-text screening stage. The first one was a misunderstanding regarding the exclusion of studies which reported on general occupational or social functioning and adjustment (see item iii of the exclusion criteria), leading to the incorrect inclusion of two studies. The second discrepancy related to overlooking a study’s sample characteristics (i.e., not being recent onset psychosis), which led to that study’s incorrect inclusion. These discrepancies were discussed between the two raters and resolved, and the eligibility criteria were refined to reduce ambiguity.

Relevant studies incorporating quantitative (*n* = 12) and qualitative (*n* = 2) methods and published between 2004 and 2025 were identified. See Table 2 for the characteristics of the quantitative studies and Table 3 for the characteristics of the qualitative studies. We did not identify any eligible case reports or case studies.

**Table 2:** Key characteristics of the included quantitative studies

Author(s) & Year	Country	Design	Aims	Sampling Strategy	Sample Size	Sample Characteristics	Diagnosis	Psychosis Measure
Fedyszyn et al. 2011 [86]	Australia	Retrospective medical record audit of a cohort	To examine the characteristics of suicide attempts in young people during the first 18 months of First Episode Psychosis treatment	Participants were part of the Early Psychosis Prevention and Intervention Centre (EPPIC) Service	N = 76	Mean age = 19.2 years (*SD* = 2.6) Age range = 15-24 years Gender = 46.6% male	A database with information about individuals’ diagnosis who were accepted into the EPPIC Service	Structured file audit tools used to extract participant demographic, clinical, and treatment information
Fekih-Romdhane et al. 2023 [87]	Tunisia	Longitudinal prospective cohort (1-year follow-up)	1. To examine suicide risk in Ultra-High Risk (UHR) and First Episode Psychosis (FEP) participants. 2. To evaluate the development and correlates of suicidal ideation over 12 months in the UHR group exclusively	Participants were recruited from the Tunisian eaRly Interventionof Psychosis (TRIP) study at the Tunisian Centre of Early Intervention in Psychosis	N = 68	Ultra-High Risk of Psychosis = 35 Mean age = 22.8 years (*SD* = 4.0) Age range = 16-35 years Gender = 45.7% male First Episode Psychosis = 33 Mean age = 27.3 years (*SD* = 4.8) Age range = 16-35 years Gender = 63.6% male	Comprehensive Assessment of At-Risk Mental States (CAARMS; Yung et al. 2005) criteria for UHR or FEP	Comprehensive Assessment of At-Risk Mental States (CAARMS; Yung et al. 2005). Positive and Negative Syndrome Scale (PANSS; Kay et al. 1987)
Haining et al. 2021 [88]	UK	Cross-sectional	To examine the prevalence of suicidality and self-harm and identify predictors of current suicidal ideation in people at clinically high risk of psychosis (CHR-P), in comparison with First Episode Psychosis (FEP), non-CHR-P (CHR-N), and healthy controls (HC)	Participants were recruited for the Youth Mental Health Riskand Resilience (YouR) longitudinal study	N = 245. n (CHR-P) = 130. n (FEP) = 15. n (CHR-N) = 47. n (HC) = 53	CHR-P: Mean age = 21.6 years (*SD* = 4.3) Age range = not reported Gender = 72.3% female. FEP: Mean age = 23.7 years (*SD* = 4.8) Age range = not reported Gender = 66.7% female. CHR-N: Mean age = 22.9 years (*SD* = 4.8) Age range = not reported Gender = 63.8% female. HC: Mean age = 22.4 years (*SD* = 3.4) Age range = not reported Gender = 67.9% female	Positive scale of the Comprehensive Assessment of At-Risk Mental States (CAARMS; Yung et al. 2005). Cognitive Disturbances (COGDIS) and Cognitive-Perceptive Basic Symptoms (COPER) items of the Schizophrenia Proneness Instrument, Adult version (SPI-A; F. Schultze-Lutter et al. 2007)	Positive scale of the Comprehensive Assessment of At-Risk Mental States (CAARMS; Yung et al. 2005). Cognitive Disturbances (COGDIS) and Cognitive- Perceptive Basic Symptoms (COPER) items of the Schizophrenia Proneness Instrument, Adult version (SPI-A; F. Schultze-Lutter et al. 2007)
Heelis et al. 2016 [89]	UK	Cross-sectional	To test the hypothesis of the Interpersonal-Psychological Theory of Suicide (IPTS) that feelings of not belonging, beliefs about being a burden, and capability to attempt suicide increased the likelihood of suicide attempt in people with first episode psychosis (FEP)	Purposive sampling of individuals with FEP from a National Health Service (NHS) Early Intervention Service	N = 45	Mean age = 24.9 years (*SD* = 4.7) Age range = 16-35 years Gender = 89% male	FEP determined by participants' psychiatrists and defined by the International Classification of Diseases 10th revision (World Health Organization, 1992)	Psychosis severity was not measured
Pelizza et al. 2020b [90]	Italy	Longitudinal prospective cohort (3-year follow-up)	1. To examine prevalence and incidence of suicide attempts, suicidal thinking and suicide deaths in First Episode Psychosis (FEP) compared to non-FEP participants 2. To examine correlations of suicidal ideation with baseline sociodemographic and psychopathological predictors. 3. To monitor the stability of suicidal thinking in FEP	The participants were part of the Reggio Emilia At-Risk Mental States (ReARMS) project	N = 142	Mean age = 23.2 years (*SD* = 5.6) Age range = 13-35 years Gender = 69% male	Comprehensive Assessment of At-Risk Mental States (CAARMS; Yung et al. 2005) criteria for FEP and <2 years duration of untreated psychosis	Comprehensive Assessment of At-Risk Mental States (CAARMS; Yung et al. 2005)
Robinson et al. 2010 [91]	Australia	Longitudinal prospective cohort (7.4-year follow-up)	1 To examine the prevalence of, and risk factors for, suicide attempts in a first episode psychosis (FEP) 2 To investigate differences between people with single and multiple suicide attempts	Participants were part of the Early Psychosis Prevention and Intervention Centre (EPPIC) Service which has a catchment area of ~800,000 people	N = 413	Mean age = 21.8 years (*SD* = 3.5) Age range = 15-30 years Gender = 70.9% male	DSM-III-R (American Psychiatric Association, 1987; 1995) and DSM-IV (American Psychiatric Association, 1994) diagnosis of a psychoticdisorder (schizophrenia, schizophreniform disorder, schizoaffective disorder, delusional disorder, bipolar psychoticdisorder, major depressive disorder with psychotic features,brief reactive psychosis/brief psychosis and psychosis nototherwise specified)	T1: Royal Park Multidiagnostic Instrument for Psychosis (RPMIP; McGorry et al. 1990a; b) T2: Brief Psychiatric Rating Scale (BPRS; Overall & Gorham, 1962; McGorry et al. 1988; Lukoff et al. 1986). Schedule for the Assessment of Negative Symptoms (SANS; Andreasen, 1982)
Serra-Arumi et al. 2023 [92]	Spain	Cross-sectional	1. To compare perceived social support between participants with FEP and healthy controls. 2. To examine sex differences regarding perceived social support in patients with FEP and healthy controls. 3. To explore which sociodemographic, clinical and psychosocial variables were related to perceived social support in the onset of FEP	FEP participants were recruited form mental healthcareunits at the Parc Sanitari Sant Joan de D’eu and the Hospital MaternoinfantilSant Joan de D’eu. Healthy controls were recruited from the general population through the social networks of the hospital and the research team	N = 146.n (FEP) = 76.n (healthy controls) = 70	Mean age: FEP = 25.6 years (*SD* = 8.7) Age range = 13-46 years Healthy controls = 24.7 years (*SD* = 8.6) Age range = 13-47 years Gender: FEP = 68.4% male Healthy controls = 71.4% male	FEP was defined as experiences delusions, hallucinations, disorganised language, catatonic or disorganised behaviour, and negative symptoms (e.g., alogia, abulia, affective flattening) for at least one week and less than five years	Positive and Negative Syndrome Scale (PANSS; Kay et al. 1987)
Tarrier et al. 2004 [93]	UK	Cross-sectional	1. To investigate the presenceof suicidal ideation and history of suicide attempts in people with recent onset schizophrenia 2. To examine the relationship of these proxy measures of suicidal tendency to participants' clinical symptoms, self-esteem, and interpersonal environment	Medical records from four NHS trusts were screened forpotentially eligible participants	N = 59	Mean age = 27.2 years (*SD* = 7.6) Age range = 18-48 years Gender = 74.4% male	DSM-IV criteria for schizophrenia, schizophreniform, or schizoaffective disorder	Positive and Negative Syndrome Scale (PANSS; Kay et al. 1987)
Tarrier et al. 2007 [94]	UK	Cross-sectional	To examine the effects and consequences of a first episode of psychosis (FEP). It was hypothesised that suicide behaviour would be associated with the negative consequences of psychosis and PTSD	Participants were recruited from psychiatric mental health hospital wards in Manchester, UK	N = 35	Mean age = 24.9 years (*SD* = 6.3) Age range = not reported Gender = 71% male	A member of the participant's inpatient care team (e.g., key worker) confirmed FEP diagnosis upon referral to the study	Positive and Negative Syndrome Scale (PANSS; Kay et al. 1987)
Wastler et al. 2020 [95]	USA	Cross-sectional	To test the applicability of the Interpersonal-Psychological Theory of Suicide (IPTS) to FEP	Participants were recruited from an uncontrolled trialof coordinated care for FEP. Baseline data were used	N = 39	Mean age = 21.6 years (*SD* = 4.1)Age range = not reported Gender = 77% male	Structured Clinical Interview for the DSM-IV-TR (First et al. 2002)	Positive and Negative Syndrome Scale (PANSS; Kay et al. 1987)
Wastler et al. 2025 [96]	USA	Cross-sectional and longitudinal prospective cohort (using Ecological Momentary Assessment [EMA])	To examine the perceived reasons for thinking about suicide of individuals with first-episode psychosis (FEP). Participants completed baseline clinical interviews and self-report measures followed by 28 days of EMA	Participants were recruited from the Ohio State University Early Psychosis Intervention Center (EPICENTER; Breitborde et al. 2015)	N = 46	Mean age = 23.6 (*SD* = 3.62) Age range = 18-35 years Gender = 52.2% male First Episode	Structured Clinical Interview for schizophrenia spectrum disorder or affective disorder with psychotic features for DSM-V (SCID; First et al. 2016)	Structured Clinical Interview for schizophrenia spectrum disorder or affective disorder with psychotic features for DSM-V (SCID; First et al. 2016)
Xu et al. 2016 [97]	Switzerland	Cross-sectional	Hypotheses: 1. Increased self-labelling as being ‘mentally ill’ and stigma stress will be associated with suicidal ideation. 2. Low social isolation and self-esteem will mediate the relationship between self-labelling and stigma stress andsuicidal ideation. Stigma stress is defined as a stigma-related harm which is perceived to exceed individual’s resources to cope with the harm	Participants were taking part in the ZInEP early recognition project	N = 172	Mean age = 21.4 years (*SD* = 5.8) Age range = 13-35 years Gender = 59.3% male	Schizophrenia Proneness Interview (SPI; Schultze-Lutter et al. 2007; Schultze-Lutter & Koch, 2010) to assess high-risk status. Structured Interview for Prodromal Syndromes (McGlashan et al. 2001; Miller et al. 2003) to assess ultra high-risk status	Positive and Negative Syndrome Scale (PANSS; Kay et al. 1987)

Author(s) & Year	Type of Suicidality	Suicidality Measures	**Type of Social Connectedness/Disconnectedness***(based on the SoCaD framework)*	Social Connectedness/Disconnectedness Measure	Analysis	Key Findings
Fedyszyn et al. 2011 [86]	Suicide attempts	Review of assessment and discharge reports, notes from emergency rooms, and suicide risk assessment forms; inspection of case notes related to the suicide attempt. Each suicide attempt was assessed with the Classification Algorithm for Determination of Suicide Attempt and Suicide (CAD-SAS). World Health Organization (WHO) Life Chart (LCS; WHO, 1992) measured suicide attempts severity. 62.8% of the sample had made suicide attempts of mild severity, 25.5% if moderate, and 11.5% of high severity	Precipitating factors of suicide attempts (e.g., relationship/family difficulties; interpersonal problems)	Suicide attempt precipitating factors were extracted from clinical notes including key precipitants of suicide attempts, as reported by the participant, using a structured file audit tool. The social connectedness/disconnectedness items were not specified	Chi-squared test was used for categorical data. Continuous data were analysed using independent *t-*test	Frequency data indicated that approximately a third of suicide attempts were precipitated by interpersonal issues (e.g., relationship problems, break up, peer conflict, family discord)
Fekih-Romdhane et al. 2023 [87]	Suicidal ideation and suicide attempt history	Suicidality/self-harm item 7.3 of the Comprehensive Assessment of At-Risk Mental States (CAARMS; Yung et al. 2005). 7-point item assessing suicidal ideation and self-injurious acts. ≥2 score indicates at least passive, occasional suicidal thoughts or ideas of self-harm, but no active suicidal plans or acts. 60% of the UHR sample and 54.5% of the FEP sample had a score ≥ 2. No significant difference in suicidal ideation between FEP and UHR groups was reported at baseline. Suicide attempt history was obtained from participants’ clinical records	Perceived social support	Multidimensional Scale of Perceived Social Support (MSPSS) – perceived adequacy of social support from friends, family, and significant others (Zimet et al. 1988)	Pearson correlation coefficients were used for continuous variables. Repeated measures ANOVA taking the suicide score at baseline, after 6 and 12 months as the dependent variable. Duration of illness, self-esteem, social support and total PANSS scores were controlled for. Partial eta squared was calculated to estimate effect size	In the UHR group, increase in PANSS scores at 1-year follow-up was associated with increased suicidal ideation (*p*=0.048) when controlling for social support, self-esteem, duration of untreated psychosis, and PANSS scores. Social support at baseline and follow-up was not significantly associated with lifetime suicide attempts and suicidal ideation (*p* > 0.05) in the UHR group
Haining et al. 2021 [88]	Suicidal ideation	Six-item suicidality scale of the Mini-International Neuropsychiatric Interview (MINI; Sheehan et al. 1998) and questions contained in the CAARMS (Yung et al. 2005) suicidality and self-harm sub-scale to measure past-month and lifetime suicidal ideation and attempts. Lifetime suicide attempts were significantly more prominent in CHR-P (29.2%) and FEP (60.0%) compared to CHR-N (8.5%) and HC (0%) participants	Perceived social support	Significant Others Scale (SOS; Power et al. 1988) measured two domains of social support from others (i.e., emotional [e.g., someone to trust/talk to frankly] and practical [e.g., provision of advice/suggestions]).	Group differences were assessed with Kruskal-Wallis H and Chi-square tests. Multivariable logistic regression was used for suicidal ideation predictors testing in the CHR-P group.	Social support from others was not identified as a significant predictor of suicidal ideation (*p* > 0.05) in the CHR-P group
Heelis et al. 2016 [89]	Suicidal ideation	Columbia-Suicide Severity Rating Scale (C-SSRS; Posner et al. 2011) in the past six months. 67% of the sample reported previous suicidal ideation. Participants with suicide attempt history had attempted suicide 3.14 times on average	Perceived burdensomeness and thwarted belongingness (feeling isolated and disconnected from others)	Responsibility to Family subscale of the Reasons for Living Inventory (RLI-RF; Linehan, Goodstein, Neilsen, & Chiles, 1983). Basic Need Satisfaction in Life Scale (BNS-LS; Johnston & Finney, 2010)	One-way ANOVAs	All participants perceived themselves to be a burden and did not feel that they belonged (measured by RLI-RF). Interpersonal-Psychological Theory of Suicide (IPTS) variables did not differentiate between participants with a history of suicidal ideation, suicide attempts, and no suicidal experiences (*p* > 0.05)
Pelizza et al. 2020b [90]	Suicidal ideation	Beck Depression Inventory (BDI), item 9 (“Suicidal thoughts or wishes” experienced in the past two weeks)	Perceived quality of social relationships	World Health Organization Quality Of Life scale - Brief version (WHOQOL-BREF) – ‘social relations’ domain which captures satisfaction with personal relationships, support received from friends, and sex life)	Correlation coefficients examined relationships between variables. Chi-square tests were used for categorical variables, and Mann-Whitney U was performed to compare continuous Variables	The BDI item 9 score which assessed suicidal thoughts/wishes was significantly negatively correlated with the ‘social relations’ domain score of WHOQOL-BREF. Suicidal ideation was related to perceived poor social relationships quality in people with FEP. However, these results are based on baseline data.
Robinson et al. 2010 [91]	Suicide attempts	World Health Organization (WHO) Life Chart (LCS; WHO, 1992): suicidal thoughts in the past two years (not at all, occasionally, often); suicide attempts (no/yes); number of suicide attempts, and severity of suicide attempts that caused most injury (mild/hardly an injury or none, moderate/some injury; severe/almost died or serious lasting injury). 21.6% of the sample had one or more suicide attempts	Premorbid social isolation or withdrawal (presence/absence prior to presentation)	Royal Park Multidiagnostic Instrument for Psychosis (RPMIP; McGorry et al. 1990)	Univariate binary logistic regression to assess associations between suicide predictors and suicide attempts	The associations between social isolation and withdrawal and suicide attempts were not significant (*p* > 0.05). There was no statistically significant difference in social isolation and withdrawal between participants who had not attempted suicide and those who attempted suicide one or more times. These results are based on baseline data.
Serra-Arumi et al. 2023 [92]	Suicide risk	Suicide Risk Scale of Plutchik (SRSP; Plutchik et al. 1989; Rubio et al. 1998) including ‘yes/no’ response questions about suicide attempts history, the strength of suicidal impulses, sleep problems, feelings of depression, worthlessness, and hopelessness	Perceived social support	Duke-UNK Functional Social Support Questionnaire (DUKE-UNK; Bellón Saameño et al. 1996; Broadhead et al. 1988), including two subscales: ‘Confidant support’ and ‘Affective support’. Confidant support includes seven items on the possibility of having people to communicate with. Affective support includes four items on receiving demonstrations of love, care affection, and empathy from others	Categorical variables were analysed using the Chi-square tests and continuous variables were compared using *t*-tests. Linear regression examined the influence of relevant variables on perceived social support for FEP participants	FEP participants had significantly higher suicide risk (*p* < 0.001) and lower perceived social support scores (*p* = 0.003) compared to the healthy controls. Linear regression analysis including perceived affective support showed that suicide risk was the only significant predictor variable. The model explained 12.9% of the variance in perceived affective support in people with FEP. Lower suicide risk was the only significant indicator for higher levels of perceived affective support
Tarrier et al. 2004 [93]	Suicidal ideation, and suicide attempt history (which created a composite suicide risk score)	Beck Scale for Suicide Ideation (BSS; Beck 1991) including past-week ideation and lifetime attempts; possible range 0–25. Sample Mean = 4.1, *SD* = 7.2. 19% had made one suicide attempt, 27.6% had made two or more attempts	Social isolation and perceived social support	Modified Self-Evaluation and Social Support for Schizophrenia (SESS-sv) Interview and Scales (Humphreys et al. 2001; Barrowclough et al. 2003), including social/recreational, occupational, relationships, parenting, and homemaking domains. Questions involve both perceived competence and commitment in each domain	Regression path analysis using a composite suicide ideation and past attempt frequency metric. Chi-squared tests for categorical variables assessed differences between participants with and without past suicide attempts	The path analysis showed that positive symptoms of psychosis were associated with social isolation which, in turn, was associated with hopelessness (a proxy measure of suicide-related experiences). Hopelessness, was directly associated with medium (*p* = 0.03) and high (*p* = 0.05) suicide risk, implying a mediation pathway (i.e., positive psychotic symptoms → social isolation → hopelessness → increased suicide risk)
Tarrier et al. 2007 [94]	Suicidal ideation and suicide attempts	Participants were asked: 1. whether they had experienced suicidal ideation as a result of psychosis, and to rate the severity of the experience; 2. if they had attempted suicide since their psychosis onset; and 3. the reasons for attempting suicide. 43.8% of the sample experienced current suicidality due to psychosis, and for 28.1% it was severe	Social exclusion	Semi-structured interview examining the consequences of psychosis, including social exclusion. Each consequence was scored on being absent or present and, if present, then rated as being of mild-to-moderate severity [1] or severe [2]	*T*-tests for continuous and Chi-squared tests for categorical variables were used	Positive symptoms of psychosis were associated with social exclusion (*p* = 0.01). There were no significant associations between suicidal ideation or suicide attempts and experiences of social exclusion (*p* > 0.05). Half of the participants reported experiences of social exclusion, and just under half reported current suicidal ideation
Wastler et al. 2020 [95]	Suicidal ideation	Columbia Suicide Severity Rating Scale (C-SSRS; Posner et al. 2011) - five suicidal ideation items (possible range: 0–5. Sample Mean = 1.61, *SD* = 2.04)	Perceived burdensomeness, thwarted belongingness (feeling isolated and disconnected from others)	Interpersonal Needs Questionnaire (INQ; Joiner, van Orden, Witte, & Rudd, 2009) measured burden someness and thwarted belongingness	Regression analysis	Participants with recent suicidal ideation reported higher levels of perceived burdensomeness (*p* = 0.01) and thwarted belongingness (*p* = 0.01) compared to participants without recent suicidal ideation. This suggested that burdensomeness and belongingness differentiated between individuals with and without suicidal ideation
Wastler et al. 2025 [96]	Lifetime suicidal ideation and reasons for thinking about suicide were measured at baseline. In addition, EMA items assessed real-time suicidal ideation and reasons for thinking about suicide	Self-Injurious Thoughts and Behaviours Interview-Revised (SITBI-R; Fox et al. 2020; Nock et al. 2007) – measures lifetime experiences. Item 11 of the Suicide Attempt Self-Injury Interview (SASII) assessed current reasons for thinking about suicide (Linehan et al. 2006). Four previously validated items were used to assess suicidal ideation during EMA (Forkmann et al. 2018): How much have you … felt that life is not worth living; felt that there are more reasons to die than live; wanted to die; and thought about taking your life. Each item was rated on a 0–4 Likert scale (Not at all-Very Much)	Feelings of aloneness, emptiness, isolation	Item 11 from the Suicide Attempt Self- Injury Interview (SASII) assessed reasons for thinking about suicide (Linehan et al. 2006) This item includes 29 reasons for injuring self/attempting suicide. The study modified these instructions to have participants indicate all the reasons why they thought about suicide in their lifetime. Participants were also provided with an open-text ‘other’ option and were asked to select all items that contributed to their suicidal ideation	Descriptive statistics examined frequencies of each reason for thinking about suicide during baseline and EMA. For EMA data, percentages were calculated out of the total number of surveys (i.e., the number of instances of suicidal ideation during EMA) and the total number of participants (i.e., the number of participants who endorsed suicidal ideation during EMA)	82.6% of the participants reported lifetime suicidal ideation, 30.4% reported lifetime suicidal behaviour. Some of the lifetime reasons for thinking about suicide reported at baseline included to relieve feelings of aloneness, emptiness or isolation (57.9%), to stop hearing things (26.3%), to die (44.7%), and ‘other’ (18.4%, which included feelings of paranoia, burdensomeness, and to stop having distorted thoughts/delusions). EMA reasons for thinking about suicide included psychosis (e.g., auditory, visual hallucinations, paranoia), (16.3% of the 566 instances of suicidal ideation). Feelings of aloneness, emptiness, and isolation were not reported to contribute to suicidal ideation in the EMA
Xu et al. 2016 [97]	Suicidal ideation	Suicidality item of the Hamilton Rating Scale for Depression (HRSD; Hamilton, 1960) which measure experiences in the past week	Social isolation	Social isolation was assessed by the five-item sub-scale (e.g., “social isolation”, “social rejection”, “being ignored”) of the Survey of Recent Life Experiences (SRLE; Kohn and Macdonald, 1992)	Correlation coefficients examined relationships between predictor and mediator variables. Path analysis examined direct and mediation effects	A significant positive correlation coefficient between social isolation and suicidal ideation. Greater stigma stress was related to social isolation which, in turn, was associated with low self-esteem, depression and suicidal ideation. Social isolation fully mediated the relationship between stigma stress and suicidal ideation

**Table 3:** Key characteristics of the included qualitative studies

Author(s) & Year	Country	Design	Aims	Sampling Strategy	Sample Size	Sample Characteristics	Diagnosis	Psychosis Measure
Gajwani et al. 2018 [98]	UK	Qualitative cross-sectional	1. To examine the meaning of suicide attempts for young men with first episode psychosis. 2. To explore young men’s experience of emerging psychosis and its relation to the suicide attempt	Purposive sampling of individuals with psychosis accessing NHS Early Intervention Services	N = 7	Mean age = 22.8 years (*SD* = 3.5) Age range = 18-35 years Gender = 100% male First Episode Psychosis = 100%	International Statistical Classification of Diseases and Related Health Problems (ICD)-10 criteria for Schizophrenia or related disorders (F20, 22, 23; World Health Organization, 1992)	Psychosis severity was not measured
Sandhu et al. 2013 [99]	UK	Qualitative cross-sectional	To examine the experience and phenomenological features of post-psychotic depression in FEP	Participants with FEP were purposively recruited from South Birmingham NHS Early Intervention Service	N = 8	Mean age = 25.4 yearsAge range = 18-35 years Gender = 62.5 % males First Episode Psychosis = 100%	ICD-10 criteria for schizophrenia and post-schizophrenic depression (F20 and F20.4; World Health Organization, 2007)	Psychosis severity was not measured

Author(s) & Year	Type of Suicidality	Suicidality Measure	**Type of Social Connectedness/Disconnectedness** *(based on SoCaD framework)*	Social Connectedness/Disconnectedness Indicant	Data Elicitation Method	Analysis	Key Findings
Gajwani et al. 2018 [98]	Suicidal ideation	Semi-structured, individual interviews. Participants without current risk of self-harm but who had attempted suicide in the last 24 months that has led to medical intervention were included	Social isolation, aloneness, loneliness, social withdrawal	Qualitative interview	Semi-structured, individual interviews	Interpretative Phenomenological Analysis (IPA)	Isolation, loneliness and withdrawal from society were perceived as profound sources of distress. Social isolation was related to active social avoidance or feeling excluded by others. Participants often described themselves as “outcasts”: ”*I was spending a lot of time alone, smoking a lot of cannabis. I generally felt quite cut off from the rest of the world … because of my feelings of loneliness, […] I felt that life – don’t know, life was just very difficult … and so I thought of various ways of committing suicide.*”
Sandhu et al. 2013 [99]	Suicidal ideation	Unstructured, individual interviews	Social isolation, loneliness, withdrawal	Qualitative interview	Photo-elicitation with unstructured interviews. Participants took photographs to record their real-life experiences. The images were used as prompts for discussions in interviews	Framework analysis	Reduced social interactions strengthened feelings of loneliness, deliberate avoidance of social situations, and thoughts about suicide: *“It’s a feeling of worthlessness, feeling of no hope, feeling of you’re useless to anything, anyone … you got nothing to look forward to, it’s all taken away from you. When I get really bad, I just feel like I just wanna get a knife and just slash my wrists because it’s that’s bad … Sometimes I always think like that, just do it, finish it, just finish it because there’s nothing left for you, there’s nothing left than a life of misery.”* Furthermore, participants reported that their family and friends became less understanding and more irritated with participants’ lack of engagement in activities. Participants felt like a burden on parents and partners: *“They [family] treat me like I’m just there, just a liability, they don’t understand me, they kind of like brush me off. It’s a bit like, you know the kid’s book ‘‘Where’s Wally?’’ It’s like I’m the different one, I feel like I’m Wally.”*

Six studies were conducted in the UK [88, 89, 93, 94, 98, 99], two in the USA [95, 96], two in Australia [86, 91], one in Tunisia [87], one in Italy [90], one in Spain [92], and one in Switzerland [97], (see Tables 2 and 3).

Of the 12 quantitatively designed studies, four used longitudinal survey designs [87, 90, 91, 96], seven used cross-sectional designs [88, 89, 92–95, 97], and one was a retrospective clinical record cohort study [86]. The length of follow-up in the four longitudinal studies was 28 days [96], one year [87], three years [90], and 7.4 years [91]. Sample sizes across the quantitative studies ranged between 35 and 413 participants (*Mean* = 123.8; *SD* = 112.2; *Median* = 72). The two qualitative studies were cross-sectional and used Interpretative Phenomenological Analysis (IPA [98]) and Framework Analysis [99])] with sample sizes of 7 and 8, respectively (see Tables 2 and 3).

Most studies (*n* = 10) included samples of participants with FEP [86, 89–92, 94–96, 98, 99]. Two studies included mixed samples of participants with FEP and UHR [87] and FEP, CHR of psychosis, non-CHR of psychosis (characterised as having psychiatric comorbidities but not fulfilling criteria for CHR of psychosis) and healthy controls [88]. Relevant results in these two studies were reported for the UHR [87] and the CHR for psychosis [88] participant groups. One study included participants experiencing recent onset schizophrenia, operationalised by the authors as a maximum ‘illness’ duration of 3 years [93], and one study included participants at risk of developing psychosis (i.e., ARMS [97]), (see Tables 2 and 3).

#### Methods of ascertaining psychosis diagnosis and measuring the severity of psychosis experiences

Seven studies used psychiatric diagnostic tools for ascertaining psychosis in their samples (see Table 2): the Diagnostic and Statistical Manual of Mental Health Disorders, 4^th^ edition (DSM-IV [100]); DSM-IV-TR [91, 93, 95, 96, 101]), and the International Statistical Classification of Diseases and Related Health Problems, 10th revision (ICD-10 [89, 98, 99, 102]). Four studies used other methods (e.g., clinical symptom assessments by researchers or health professionals, information from medical records [86–88, 94]). In addition, 11 studies assessed the severity of psychosis symptoms using validated measures, such as the Positive and Negative Syndrome Scale (PANSS [87, 92–95, 97]), Comprehensive Assessment of at Risk Mental States (CAARMS [87, 88, 90]), Structured Clinical Interview for DSM-IV and V (SCID [96]), Royal Park Multi-diagnostic Instrument for Psychosis (RPMIP [91]). One study used a non-validated measure which was an audit tool to extract relevant participant clinical information [86]. The two qualitative studies [98, 99] and one cross-sectional quantitative study [89] did not use a psychosis severity measure (see Tables 2 and 3).

#### Methods of measuring suicidal experiences

Most studies (*n* = 8) reported findings in relation to experiences of suicidal ideation [88–90, 95–99]. Two studies examined suicide attempts [86, 91]. Two studies investigated both suicidal ideation and suicide attempts [87, 94], and two studies examined suicide risk [92, 93]. In Serra-Arumi et al.’s study [92], ‘suicide risk’ was operationalised as a composite score on a scale measuring history of suicide attempts, strength of suicidal impulses, depression, sleep problems, and sense of worthlessness and hopelessness. In Tarrier et al.’s study [93], ‘suicide risk’ was operationalised as suicidal ideation and suicide attempt history.

Suicidal experiences in the included studies were assessed using different approaches and scales. Only four studies used validated, suicidality-specific measures, such as the Columbia-Suicide Severity Rating Scale (C-SSRS [89]), Suicide Risk Scale of Plutchik (SRSP [92]), Beck Scale for Suicide Ideation (BSS [93]), and Self-Injurious Thoughts and Behaviours Interview-Revised (SITBI-R [96]).

Eight studies used one or more items measuring suicidal experiences from other validated measures, for example, the suicidality and self-harm items from the Comprehensive Assessment of At-Risk Mental States (CAARMS [87, 88]), the suicidality module of the Mini-International Neuropsychiatric Interview (MINI [88]), item 9 from the Beck Depression Inventory (BDI) which assesses suicidal thoughts or wishes [90], suicide-related items from the World Health Organization Life Chart Schedule (WHO LCS [86, 91]), the suicidal ideation items from the Columbia-Suicide Severity Rating Scale (C-SSRS [95]), four Ecological Momentary Assessment (EMA) items assessing suicidal ideation severity [96], one item from the Suicide Attempt Self-Injury Interview (SASII) measuring reasons for thinking about suicide [96], and the suicidality item from the Hamilton Rating Scale for Depression (HRSD [97]. Of note, three studies used a combination of two suicide measures, i.e., SITBI-R and SASII, in addition to EMA items for suicidal ideation severity [95], WHO LCS and a suicide attempts classification system [86], and MINI and CAARMS [88], (see Table 2).

One study used a non-validated measure including three self-report questions about experiences of suicidal ideation, suicide attempts, and the reasons for attempting suicide [94]. Two studies obtained information about suicide attempt history from participants’ clinical records [87] or reviewed clinical notes about suicidal experiences and categorised them using an algorithm for suicide attempts and suicide deaths [86]. Two studies used qualitative interview questions about suicidal experiences [98, 99], (see Table 3).

#### Methods of measuring perceptions of social connectedness and social disconnectedness

To assess social connectedness and/or disconnectedness, four studies used validated connectedness-specific questionnaire measures, namely, the Multidimensional Scale of Perceived Social Support (MSPSS [87]), the Significant Others Scale (SOS [88]), the Modified Self-Evaluation and Social Support for Schizophrenia Interview and Scales (SESS-sv [93]), the Interpersonal Needs Questionnaire (INQ [95]).

Eight studies used non-validated measures, namely, information from participant clinical case notes [86], sub-scales from other validated questionnaire measures (i.e., Reasons for Living Inventory (RFI) – ‘responsibility to family’ sub-scale [89]; World Health Organization Quality of Life scale - Brief version (WHOQOL-BREF) – ‘social relationships’ domain [90], Duke-UNK Functional Social Support Questionnaire (DUKE-UNK) – ‘confidant support’ and ‘affective support’ sub-scales [92], and the Survey of Recent Life Experiences (SRLE) – ‘social isolation’, ‘social rejection’, ‘being ignored’ sub-scales [97], or an interview measure (i.e., Royal Park Multidiagnostic Instrument for Psychosis (RPMIP [91])), a semi-structured interview examining the presence or absence of consequences of psychosis, including social exclusion [94], and Ecological Momentary Assessment (EMA) items assessing reasons for thinking about suicide (e.g., aloneness, isolation [96]), (see Table 2).

Two studies used qualitative interviews [98, 99] to examine perceptions of social connectedness/disconnectedness (see Table 3). Most of the included studies used non-validated measures or single items from validated measures which is problematic for ascertaining validity and sensitivity of the reported perceptions of social connectedness and disconnectedness.

### Findings from studies using quantitative designs

The SoCaD framework (see Fig. 1) was used to structure the relevant evidence from the included quantitative studies in relation to the six domains of perceptions of social connectedness and social disconnectedness. There were findings aligning with only three elements within the framework.

1. Not having valued supportive relationships with other people.

Six studies contributed evidence for this domain with samples sizes ranging between 46 and 245 participants (*Mean* = 120.5, *SD* = 73.3) and various suicide-related indicants, such as suicidal ideation, suicide attempts, and suicide risk [86–88, 90, 92, 96]. A retrospective cohort study, with a mean period of observation of approximately 1 year and 7 months (i.e., 574.5 days) reported an increased frequency of suicide attempts among participants experiencing interpersonal issues, such as interpersonal relationship difficulties, including relationship ending, conflict with peers and/or family, although it should be noted that details about the nature of these problems were not provided [86]. The study found that interpersonal issues could precipitate suicide attempts recorded during the retrospective observation period. This occurred for around 35% of the attempts and was the most frequent precipitant recorded. In comparison, distress from psychotic experiences was identified as a precursor for only 16% of the attempts [86]. Similarly, nine participants (39.1% of the sample) in Wastler et al.’s study [96] reported that interpersonal problems made them think about suicide.

A three-year longitudinal study examined the perceived quality of social relationships using a measure including items assessing satisfaction with received support from friends, personal relationships, and satisfaction with sex life. The study found that suicidal ideation was significantly correlated with perceived poor social relationship quality [90]. However, the study did not examine any differential effects of received support from friends, quality of personal relationships, and satisfaction with sex life on suicidal experiences. It should also be noted that even though this study was carried out over three years, there was only relevant baseline data that contributed to this finding.

Notable for null findings, a one-year longitudinal study found no significant relationships between perceived social support (i.e., from family, friends, and significant others as an overall score), and suicidal ideation and lifetime suicide attempts [87]. Perceptions of social support did not change over time at baseline, 6 months and 12 months follow-up and were not statistically significantly different between participants with and without suicide attempt history. Similarly, there was no significant relationship between perceived social support from others and lifetime suicide attempts in a cross-sectional study [88]. This study used a measure of perceived social support from others which tapped into two constructs – emotional and practical support. However, the study did not examine these two different constructs separately.

A different cross-sectional study probed into different types of social support by examining confidant social support, i.e., having people to communicate with, and affective social support, i.e., receiving demonstrations of love, care affection, and empathy from others [92]. In contrast to the findings documented by Fekih-Romdhane et al. [87] and Haining et al. [88], perceived lack of affective support, but not confidant support, was significantly associated with suicide risk. Lower suicide risk was the only significant indicator for higher levels of perceived social support in the form of expressions of love, care affection, and empathy from others. Of note, the suicide risk measure in that study captured an amalgamation of suicide-related experiences, such as suicide attempt history, sleep problems, the strength of suicidal ‘impulses’, feelings of depression, anger, worthlessness, and hopelessness meaning that this indicant may have been more sensitive to ‘suicide risk’ but not necessarily more specific.

2. Not having a social identity or social purpose.

No quantitatively designed studies examined perceptions of having or lacking a social identity, social involvement, and social purpose in a community or society.

3. A sense of not belonging.

Seven studies contributed evidence for this domain with samples sizes ranging between 35 and 413 participants (*Mean* = 129, *SD* = 147.9), and different types of suicide-related indicants, such as past and current suicidal ideation, suicide attempt history, and reasons for thinking about suicide [89, 91, 93–97]. Related constructs to a sense of not belonging investigated by the included studies were perceived burdensomeness, social isolation, and social exclusion.

Two cross-sectional studies [89, 95] tested the Interpersonal-Psychological Theory of Suicide (IPTS [58]); in young people with first episode psychosis (FEP). These two studies used the Interpersonal Needs Questionnaire (INQ [95]), the Reasons for Living Inventory (RLI-RF [responsibility to family sub-scale] [89]), and the Basic Need Satisfaction in Life Scale (BNS-LS [89]). The INQ measures perceptions of thwarted belongingness and burdensomeness, whereas the Responsibility to Family sub-scale of the RLI-RF measures perceived sense of commitment and responsibility to family and the BNS-LS assesses satisfaction with the need for autonomy, relatedness and competence. It is unclear to what extent the RLI-RF and BNS-LS used by Heelis et al. [89] respectively map onto the concepts of thwarted belongingness and perceived burdensomeness.

Heelis et al. [89] reported that all participants in their study felt that they did not belong and were a burden to others. However, the perceived burdensomeness and thwarted belongingness variables did not differentiate between participants with a lifetime history of suicidal ideation compared to those reporting suicide attempts, and those with no suicidal experiences. In contrast, the participants with recent suicidal ideation in a study by Wastler et al. [95] reported significantly higher levels of perceived burdensomeness and thwarted belongingness compared to participants without recent suicidal ideation, which is in line with the IPTS model. One key difference between the Heelis et al.’s [89] and Wastler et al.’s [95] study was that the participants in Wastler et al.’s [95] study were experiencing recent suicidal ideation, whereas those in Heelis et al.’s [89] study had experienced long-term suicidal ideation. However, the main effects and interaction between burdensomeness and thwarted belongingness did not predict the severity of suicidal ideation in Wastler et al.’s study [95] potentially due to the insufficient sample size. That is, the synergistic effect of perceptions of burdensomeness and thwarted belongingness did not lead to increased suicidal ideation severity. These results showed that both perceived burdensomeness and thwarted belongingness differentiated between participants with and without recent suicidal ideation and may relate to the development of suicidal ideation in people experiencing psychosis [95].

Perceived social isolation was found to be an important predictor of suicidal ideation and suicide risk in people with recent onset psychosis [93, 97]. Specifically, in one cross-sectional study, using path analysis, positive psychotic symptoms were found to be associated with social isolation and hopelessness which, in turn, increased suicide risk scores (created by a composite score of suicidal ideation and suicide attempt history as measured by the Beck Scale for Suicidal Ideation (BSI [93])). Although a mediation model was not directly tested in that study, the results may imply an indirect pathway from positive psychotic symptoms to suicide risk which is mediated by perceptions of social isolation and hopelessness. One cross-sectional study which was able to test a mediation model found that perceived social isolation fully mediated the relationship between what was termed ‘stigma stress’ and suicidal ideation severity [97]. Stigma stress was operationalised as a stigma-related harm which was perceived to exceed participants’ resources to cope with the harm. Social isolation was significantly correlated with suicidal ideation [97]. However, the associations between perceived social isolation and withdrawal and suicide attempts were not significant in a longitudinal study with a follow-up period of 7.4 years [91]. It should be noted that only baseline data contributed to this finding. The relevant domains that captured indicants of social connectedness and disconnectedness in the scales used in some of these studies were obscure. For example, it was not clear which items of the Royal Park Multidiagnostic Instrument for Psychosis (RPMIP) in Robinson et al.’s study [91] measured the presence and absence of social isolation or withdrawal and which items of the Modified Self-Evaluation and Social Support for Schizophrenia (SESS-sv) Interview and Scales in Tarrier et al.’s study [93] measured perceptions of social isolation.

Social exclusion shares conceptual similarities with social isolation, but whereas social isolation can be an individual’s decision to withdraw which is not necessarily negative (e.g., needing space), or, conversely, the result of feeling judged, shamed, or not fitting in, social exclusion can be indirect/unintentional (e.g., biases, systemic inequalities) and direct/intentional (e.g., a perceived attempt to actively exclude an individual [103, 104]). A cross-sectional study examining the impact of social exclusion from others on suicidal experiences reported no significant associations between experiences of social exclusion, positive psychotic symptoms, and suicidal ideation or attempts [94]. However, positive psychotic symptom severity was significantly higher for participants who felt socially excluded, compared to those who did not feel socially excluded. The type of social exclusion examined in that study was not explained.

A more recent study examined both lifetime and in-the-moment reasons for thinking about suicide [96]. Lifetime reasons were measured cross-sectionally at baseline. In-the-moment reasons were captured using a micro-longitudinal, Ecological Momentary Assessment (EMA) design over 28 days [96]. Lifetime reasons for thinking about suicide measured at baseline included relief of feelings of aloneness, emptiness and isolation (reported by 57.9% of participants). To get away or escape (81.6%), to stop hearing things (26.3%), and to stop feelings of burdensomeness, paranoia, and having distorted thoughts/delusions (18.4%) were additional reasons for thinking about suicide. In-the-moment reasons were largely collected with predefined responses which did not capture thoughts and feelings related to social connectedness and disconnectedness. Open responses were invited but provided by only nine participants. One of these reasons-categories was labelled as ‘interpersonal reasons’ but no further information was given.

4. Perceived social value.

No quantitative studies examined perceptions of having social capital or something to offer that is valued by others or feeling like an unwanted burden and a drain on resources.

5. A sense of not mattering to others.

Only one study contributed evidence for this domain and had a sample size of 46 participants who had a lifetime history of suicidal ideation [96]. The study identified reasons for thinking about suicide which related to perceptions of not mattering, feeling invisible and not cared about. Specifically, making others understand how desperate the participant felt as a result of psychotic experiences (15.8%), wanting to communicate feelings of despair to others (23.7%) and to get help from others (17.4%) were frequently reported reasons. A reason reported in the EMA part of the study was to communicate/get help from others, endorsed by 17.4% of the participants, which echoes communicating feelings of despair to others in the baseline reasons for thinking about suicide [96].

6. Online social interactions and connections.

No quantitative studies specifically examined experiences and perceptions of social connectedness or disconnectedness in the context of online social media activity and communication.

### Findings from studies using qualitative designs

There were two qualitative studies included in this review [98, 99]. Please see Table 3 for their key characteristics. The meta-ethnography including the two relevant qualitative studies [98, 99] was conducted following Noblit and Hare’s [69] seven steps of meta-ethnography (see Methods section). The raw data from each study (i.e., participant quotes representing first order constructs) are shown in Table 4 together with second order (i.e., study authors’) and third order (i.e., reviewers’) interpretations and themes.

**Table 4:** Data extraction including first, Second and third order constructs and interpretations

Participant quotes reported in included studies (First order constructs)	Study author interpretations (Second order constructs)	Reviewers’ interpretations (Third order constructs)	Themes
”*I was spending a lot of time alone, smoking a lot of cannabis. I generally felt quite cut off from the rest of the world … because of my feelings of loneliness,* […] *I felt that life – don’t know, life was just very difficult … and so I thought of various ways of committing suicide.*” [98]	The narrative around suicidal thinking and social isolation encompassed being alone, an existential sense of loneliness and making sense of difficult experiences	Being excluded by and actively avoiding other people by choice stemmed from perceived inability to relate to others and an attempt to reduce psychological suffering. Being cut-off from the world related to feelings of loneliness and thoughts about suicide	Life being difficult, being alone, feeling lonely and isolated from others co-occurs with a kind of disconnect (feeling cut off) which feeds back into isolation and staying isolated. This spiral contributes to thoughts about suicide
*“It’s a feeling of worthlessness, feeling of no hope, feeling of you’re useless to anything, anyone … you got nothing to look forward to, it’s all taken away from you. When I get really bad, I just feel like I just wanna get a knife and just slash my wrists because it’s that’s bad … Sometimes I always think like that, just do it, finish it, just finish it because there’s nothing left for you, there’s nothing left than a life of misery.”* [99] *“They* [family] *treat me like I’m just there, just a liability, they don’t understand me, they kind of like brush me off. It’s a bit like, you know the kid’s book ‘‘It’s like I’m the different one, I feel like I’m Wally.” Where’s Wally?’’* [99]	For some participants, the sense of hopelessness was so great that suicidal ideation began to set in	Intense feelings of no hope, being worthless, not mattering to anyone, and misery led to thoughts of suicide	Pervasive sense of being a burden, useless to others, with little hope for change

The concepts identified in the two qualitative studies were similar, rather than contradictory, therefore, reciprocal synthesis was appropriate. Two themes were constructed, based on the second order and third order interpretations, namely, 1. A continuous cycle of being alone, feeling lonely, isolated and disconnected from others; 2. Pervasive sense of being a burden, useless to others, with little hope for change.

The SoCaD framework (see Fig. 1) was used to structure the relevant evidence from the two qualitative studies in relation to the six domains of social connectedness and disconnectedness. There were findings aligning with four elements within the framework.

1. Not having valued supportive relationships with other people.

There was no qualitative evidence in relation to having or lacking valued supportive and trusting relationships with others (e.g., friends, family, partners, colleagues, peers).

2. Not having a social identity and purpose.

One study contributed evidence for this domain [99]. The study had a sample size of eight participants who were experiencing suicidal ideation.

Reduced social interactions led participants to stop being a part of activities which, in turn, amplified their sense of worthlessness, hopelessness, and lack of purpose:

It’s a feeling of worthlessness, feeling of no hope, feeling of you’re useless to anything, anyone … you got nothing to look forward to, it’s all taken away from you. When I get really bad, I just feel like I just wanna get a knife and just slash my wrists because it’s that’s bad … Sometimes I always think like that, just do it, finish it, just finish it because there’s nothing left for you, there’s nothing left than a life of misery.

These kinds of experiences further strengthened feelings of loneliness, deliberate avoidance of social situations, ultimately leading to thoughts about suicide [99]:

3. Not having a sense of belonging.

One study contributed evidence for this domain [98]. The study had a sample size of seven participants experiencing suicidal ideation. The participants in that study described experiences of social isolation stemming from two processes: 1. active avoidance of others and 2. feeling excluded by others. Active avoidance was a choice for some participants which was brought about by perceived inability to form friendships and relate to others. Other participants described a feeling of being an ‘outcast’ and excluded by others which epitomised an existential sense of loneliness that in turn led to thoughts of suicide:

I was spending a lot of time alone, smoking a lot of cannabis. I generally felt quite cut off from the rest of the world … because of my feelings of loneliness, […] I felt that life – don’t know, life was just very difficult … and so I thought of various ways of committing suicide.

4. Perceived social value.

One study contributed evidence for this domain [99]. The participants in that study described feeling like a burden to significant others, such as family: “*They [family] treat me like I’m just there, just a liability …*” and also “*feeling of you’re useless to anything, anyone …*” These experiences relate to perceptions of having no social capital and nothing to offer.

5. A sense of not mattering to others.

Only the study by Sandhu and colleagues [99] contributed evidence for this domain. One participant described a sense of being different, dismissed, irrelevant (or not mattering to) and not understood by family:

They [family], […] they don’t understand me, they kind of like brush me off. It’s a bit like, you know the kid’s book ‘‘It’s like I’m the different one, I feel like I’m Wally. Where’s Wally?

This sense was amplified by friends’ and family’s expressed frustrations at participants’ lack of engagement in daily activities, which made participants feel like a burden to others. Being dismissed was reflected in healthcare professionals not considering participants’ concerns regarding their mental health difficulties, leading to a sense of not mattering.

6. Online social interactions and connections.

No qualitative studies specifically examined experiences and perceptions of social connectedness or disconnectedness in the context of online social media activity and communication.

### Quality appraisal of the included studies

The quality appraisal ratings for the included studies are presented in Table 5.

**Table 5:** Quality appraisal of the included studies (*N* = 14)

Author(s) and year	Quality rating
Fedyszyn et al. 2011 [86]	Moderate-high
Fekih-Romdhane et al. 2023 [87]	Moderate-high
Gajwani et al. 2018 [98]	Moderate-high
Haining et al. 2021 [88]	Moderate
Heelis et al. 2016 [89]	Moderate
Pelizza et al. 2020b [90]	High
Robinson et al. 2010 [91]	Moderate-high
Sandhu et al. 2013 [99]	High
Serra-Arumi et al. 2023 [92]	Moderate-high
Tarrier et al. 2004 [93]	Low-moderate
Tarrier et al. 2007 [94]	Low-moderate
Wastler et al. 2020 [95]	Moderate-high
Wastler et al. 2025 [86]	Moderate-high
Xu et al. 2016 [97]	Moderate

There was substantial agreement on the quality scores independently rated by KH and LM (*ICC* = .91; 95% CI:. [72–91, 93–96, 98, 99]. There were discrepancies relating to the quality ratings of three studies whereby one rater had scored items assessing sample representativeness and complete outcome data conservatively which resulted in a lower overall score. The ratings for these three studies were adjusted accordingly and agreed upon.

Only the quantitative non-randomised, quantitative descriptive, and qualitative study categories of the MMAT were used in the appraisal as we did not identify any eligible quantitative randomised controlled trials and mixed methods studies (see Supplementary Table S2 for the quality assessment tool). Of note, two longitudinal studies provided relevant evidence which was cross-sectional (i.e., from baseline assessments [90, 91]). The quality ratings for those two studies reflect the methodologies and relevant data obtained, rather than the overall study designs.

The methodological quality of the quantitative studies was low-moderate to high. The quality scores of the two qualitative studies were moderate-high [98] and high [99]. No studies were of low quality.

Two studies were of low-moderate quality [93, 94]. These studies had insufficient detail regarding the participant sampling strategy and representativeness, risk of non-response bias, and completeness of the outcome data. Three studies were of moderate quality [88, 89, 97] seven were of moderate-high quality [86, 87, 91, 92, 95, 96, 98] and two studies were of high quality [90, 99]. The quality ratings did not seem to be related to the type of suicidal experiences and social connectedness and social disconnectedness measures used. That is, both higher- and lower-quality studies used validated and non-validated measures of suicidal experiences and social connectedness or social disconnectedness. However, studies of lower quality had used clinical notes/chart reviews to ascertain participants’ psychosis diagnoses. Furthermore, the domains of the SoCaD framework for which there were data, the quality rating of the studies did not appear to impact any domain more than other. Studies with different quality ratings contributed data to the SoCaD domains. The two lowest-rated studies (i.e. [93, 94]), were included in the domain with most empirical evidence – having a sense of belonging.

Overall, all quantitative studies had clear research questions and/or aims and included data that allowed the research questions/aims to be tested and addressed. However, information about the inclusion of covariates in the analyses and the representativeness of the included participants was not always available.

### Synthesis of quantitative and qualitative findings

Most of the evidence in this review was from quantitative studies adopting cross-sectional and longitudinal prospective designs, one of which used Ecological Momentary Assessment (EMA), (*n* = 12). Only two studies used qualitative approaches. The overall methodological quality of the included quantitative studies in this review was moderate and of the qualitative studies was moderate-high.

Upon examination of the factors relating to social connectedness and disconnectedness that were identified in the included studies, evidence converged across three domains of the SoCaD framework, namely, relationships with other people (*n* = 6 [86–88, 90, 92, 96], a sense of belonging (*n* = 8 [88, 89, 91, 93–97], and a sense of mattering to others (*n* = 2 [96, 99]). Only quantitative studies were included in the ‘valued supportive relationships with other people’ domain, whereas the two qualitative studies were included in the ‘having a sense of belonging’ [98] and ‘a sense of mattering to others’ [99] domains.

Evidence was sparce for two SoCaD domains, namely, ‘having no social identity and purpose’, and ‘perceived social value’. Only one study using qualitative methodology [99] provided evidence of relevance to those two domains which was not identified in any of the quantitative studies (see Table 6).

**Table 6:** Study synthesis based on the SoCaD framework

SoCaD domain	Study	Suicidal experience(s)	Quality rating
1. Valued supportive relationships with other people	Fedyszyn et al. (2011) [86],	Suicide attempts	Moderate-high
	Fekih-Romdhane et al. (2023) [87],	Suicidal ideation and attempt history	Moderate-high
	Haining et al. (2021) [88],	Suicidal ideation	Moderate
	Pelizza et al. (2020b) [90],	Suicidal ideation	High
	Serra-Arumi et al. (2023) [92],	Suicide risk	Moderate-high
	Wastler et al. (2025) [96],	Suicidal ideation/reasons for thinking about suicide	Moderate-high
2. Having a social identity and purpose	Sandhu et al. (2013) [99],	Suicidal ideation	High
3. Having a sense of belonging	Gajwani et al. (2018) [98],	Suicidal ideation	Moderate-high
	Heelis et al. (2016) [89],	Suicidal ideation	Moderate
	Tarrier et al. (2004) [93],	Suicidal ideation, and suicide attempt history	Low-moderate
	Tarrier et al. (2007) [94],	Suicidal ideation and attempts	Low-moderate
	Robinson et al. (2019) [91],	Suicide attempt	Moderate-high
	Wastler et al. (2020) [95],	Suicidal ideation	Moderate-high
	Wastler et al. (2025) [96],	Suicidal ideation/reasons for thinking about suicide	Moderate-high
	Xu et al. (2016) [97],	Suicidal ideation	Moderate
4. Perceived social value	Sandhu et al. (2013) [99],	Suicidal ideation	High
5. A sense of mattering to others	Sandhu et al. (2013) [99],	Suicidal ideation	High
	Waster et al. (2025) [96],	Suicidal ideation/reasons for thinking about suicide	Moderate-high
6. Online social interactions and connections	None	–	–

Notably, we did not identify any studies that investigated the relationships between *online* social connectedness and disconnectedness, suicidal experiences, and recent onset psychosis which represents a substantial gap in the evidence. Furthermore, none of the 14 included studies stated as a specific aim or research question the examination of experiences or perceptions of social connectedness or social disconnectedness and their relationships with suicidal experiences and psychosis in people with recent onset psychosis. That is, explorations or understanding of these relationships were not specifically formulated as objectives/aims or research questions in the studies. Rather, these factors were included in exploratory analyses or as correlates of, or risk factors for, suicidal experiences in the quantitative studies or spontaneously communicated in interviews in the qualitative studies.

## Discussion

This systematic literature review examined, for the first time, the empirical evidence for the ways in which perceptions of social connectedness and social disconnectedness can affect psychosis and suicidal experiences, specifically in people with recent onset psychosis. The evidence relating to social connectedness and disconnectedness converged across five domains of the proposed SoCaD framework (see Fig. 1 and Table 6). Despite the main aim of this review being to understand the impact of *both* online and offline social connectedness and disconnectedness, no studies were identified that specifically examined links between *online* social contexts, recent onset psychosis and suicidal experiences.

There is substantial evidence relevant to the roles of feeling connected to and supported by others in improving wellbeing and ameliorating suicidal experiences [25, 28–34, 57]. However, this review revealed somewhat mixed outcomes regarding the SoCaD domain of having valued relationships with other people which included the influence of perceived social support. Two studies (e.g. [87, 88]), reported no significant associations between social support and suicidal ideation or attempts, whereas one study [92] found that perceptions of high levels of affective social support (i.e., love, care affection, and empathy from others), but not confidant support, were related to lower suicide risk. This indicated that perceptions of social support may not always be associated with fewer or less severe suicidal experiences in people with recent onset psychosis. Discerning what it is about social support that impacts on suicidal experiences is important, however, this was beyond the scope of the included studies. Moreover, these three studies used different measures of social support, assessing perceived adequacy of social support from friends, family, and significant others (Multidimensional Scale of Perceived Social Support (MSPSS [105]), perceived practical and emotional support from significant others (e.g., spouse, partner, relative, sibling, parent, child, friend, neighbour, work colleague; Significant Others Scale (SOS [106])), and perceived affective and confidant support (Duke-UNK Functional Social Support Questionnaire (DUKE-UNK [107, 108])).

These findings resonated with a recent qualitative study by Gooding and colleagues [57] which revealed a complex interplay between suicidality, psychosis and interpersonal relationships. Participants in that study perceived interpersonal relationships as challenging due to reluctance to connect with others, actively disconnecting from others, or being left by others as a result of mental health difficulties, including psychosis and suicidality. In addition, feeling cared for, valued, acknowledged, and understood by other people, regardless of their familial relationship to the individual, was pivotal in countering suicidal thoughts and feelings [57].

Evidence in relation to the SoCaD domain ‘sense of belonging’ was provided by studies which linked perceptions of social isolation and loneliness with suicidal thoughts and attempts [89, 94–99]. This corroborates the findings of the wider literature of the deleterious impact of loneliness and social isolation on suicidal experiences in individuals with recent onset psychosis. For example, perceptions of social isolation and loneliness have been related to increased risk of suicidal experiences, such as thoughts, acts, behaviours and deaths [97, 109–114]. The findings of the qualitative study in this review using Interpretative Phenomenological Analysis (IPA [98]); further illustrated the profound psychological distress stemming from isolation and withdrawal which led to thoughts about suicide which added to the quantitative evidence for this SoCaD domain. These findings highlight the need for suicide prevention initiatives targeting social reintegration and meaningful support networks, for example, resilience-building support systems, psychoeducation, and efforts to reduce exclusion due to societal stigma around recent onset psychosis [15].

Relatedly, two studies tested the extent to which the Interpersonal-Psychological Theory of Suicide (IPTS [58]); could be applied to the experiences of suicidality of individuals with first episode psychosis (FEP [89, 95]). The IPTS includes core social disconnectedness constructs as precipitants of suicide attempts. Specifically, the theory posits that perceptions of social disconnectedness, isolation and alienation (i.e., ‘thwarted’ belongingness), feeling like a burden on others (i.e., ‘perceived burdensomeness’), and having capability to attempt suicide (acquired through increased pain tolerance, witnessing and/or experiencing adverse events, such as abuse, self-harm, suicide attempts), together, contribute to an increased likelihood of a suicide attempt [58]. Both studies that tested the IPTS [89, 95] found that perceived burdensomeness and thwarted belongingness were prevalent among participants with FEP, but only Wastler et al.’s study [95] showed a significant differentiation in these two factors between individuals with and without recent suicidal ideation. This suggests that while IPTS constructs are relevant, they may not fully capture the complexity of suicidal ideation in individuals with recent onset psychosis which necessitates a nuanced approach that considers factors that may be specific to this population. Long-term suicidal ideation may have become a consistent part of a person’s identity and a means of dealing with and regulating difficult and overwhelming emotions [115]. Furthermore, studies using Ecological Momentary Assessment (EMA) designs have captured considerable variability in perceived thwarted belongingness’ and burdensomeness within short time periods (e.g., over minutes, hours [116–118] which may have a lesser impact on long-term experiences of suicidal ideation. These results show that the IPTS may help target modifiable suicide precursors in individuals who experience different mental health problems.

The evidence for three SoCaD domains, namely, having social identity and purpose, perceived social value, and a sense of mattering to others was sparce but these domains warrant comprehensive investigation in populations with recent onset psychosis. Perceived mattering to other people, communities, and society appears to be an important determinant for positive mental health [41, 42]. Focusing on this concept in relation to suicidal experiences in people experiencing psychosis appears an important direction for future research [57].

Of note, the extent to which the social connectedness measures used by the included quantitative studies captured the construct seemed questionable and the studies did not examine the different aspects that the measures attempted to capture. The impact of perceptions of specific types of social support (e.g., care, affection) from family, friends, significant others, carers, professionals, and peers on suicidal experiences could be examined more comprehensively in populations with recent onset psychosis [92]. In relation to this, a recently published systematic literature review identified five social connectedness measures which showed robust psychometric properties (see [119], i.e., the 3-item University of California, Los Angeles loneliness scale (UCLA-3 [120]), Multidimensional Scale for Perceived Social Support (MSPSS [105]), Brief Perceived Social Support Questionnaire (F-SozU K-6 [121]), and the five- and ten-item Social Provisions Scale (SPS-5; SPS-10 [122], Lang, & Yurkowski, 2019). A limitation of that review is that it specifically focused on measures of perceived loneliness and social support [119]. The social connectedness concept is multi-faceted and contains other relevant domains as indicated in the SoCaD framework.

### Strengths and limitations

This systematic literature review has five methodological limitations that need to be considered when interpreting the findings. First, grey literature studies were not included, meaning that potentially relevant studies may have been overlooked. We chose to not include grey literature because of the lack of a peer-review process which may impugn the empirical rigour and methodological quality of such studies [123]. We also excluded papers not published in English which may have omitted perspectives and experiences of marginalised/underrepresented groups that are particularly lacking in this literature. Second, a goal of this review was to be as inclusive as possible of studies with different methodological designs (e.g., case studies, single case reports, mixed methods, qualitative studies). The breadth of methodological designs did not permit the use of a meta-analytic approach. The third limitation relates to the scope of the conceptualisation of social connectedness and disconnectedness. Specifically, we excluded structural and demographic indicants, such as social networks size, living situation and relationship status [43] which may be important determinants of suicidal experiences in people with recent onset psychosis. The fourth limitation related to the implementation of proportional independent screening, as opposed to independent screening of all records. This was a pragmatic decision, considering the large number of results identified across the four electronic databases and aligns with published guidelines for screening for systematic literature reviews [124]. The fifth limitation related to the PROSPERO protocol which did not detail the implementation of the SoCaD framework. This could have potentially introduced interpretive bias. However, the framework was developed iteratively, rather than *post hoc*, and was used as an organising scaffold during analysis and synthesis.

Despite these limitations, this review has three strengths. The first strength was the development of a theoretically informed framework – SoCaD (see Fig. 1). The framework helped organise and critically scrutinise the results and enabled systematic comparison across the included studies by mapping findings onto clearly defined domains. This facilitated identification of patterns, consistencies, and divergence within and between studies, and supported in-depth synthesis. Importantly, the framework enhanced transparency by making explicit the interpretive structure underpinning the synthesis.

The second strength was the use of a convergent explanatory design. This is an important feature because it provides in-depth understanding of complex dynamics via corroboration of quantitative and qualitative evidence [67].

The third strength is the assessment and evaluation of the methodological quality of the included studies. Quality evaluation is considered good practice in the conduct of systematic literature reviews that can prevent potential biases in the interpretation of outcomes [125, 126]. Hence, a thoroughly synthesised evaluation of the research question, the quality, methodological limitations, and risk of bias in the included articles was rigorously examined.

### Recommendations for future research

There are three considerations for future research that will advance our understanding of the relationships between perceptions of social connectedness and disconnectedness and suicidal experiences in recent onset psychosis. First, the current literature review did not find any studies that investigated *online* interactions. Examining the differential impacts of offline and online social interactions (including parasocial contacts/interactions [127]); in providing support or exacerbating mental health problems and distress is crucial. Online means of interaction and social media use have become increasingly embedded in young people’s lives [128]. This is also the case for the emerging use of generative artificial intelligence (GenAI) programmes, such as ChatGPT, as a source of mental health and psychological support [129, 130]. Despite the perceived valuable effects on mental health, there are aspects of human connection that GenAI may be unable to replicate, such as conveying human empathy and genuine care, which makes GenAI less helpful [131].

Second, mixed-methods convergent designs are needed to obtain a nuanced and comprehensive understanding of the relationships between social connectedness and disconnectedness, recent onset psychosis and suicidal experiences [132]. An example is the included study by Wastler et al. [96] which used baseline questionnaire measures and a micro-longitudinal approach, namely Ecological Momentary Assessment (EMA). In addition, studies integrating and converging qualitative insights with quantitative data would offer in-depth understanding of the lived experiences of individuals with recent onset psychosis [132].

Third, perceptions of social connectedness and disconnectedness are complex, dynamic, and can vary across time and contexts [133]. Therefore, examining variability in perceptions of connectedness and disconnectedness is an important research endeavour. Future research could use an array of convergent, fine-grained approaches that allow complex, interacting pathways to be specified and tested both in the moment and over time, in conjunction with creative qualitative data elicitation methods implementing a variety of communication modes that resonate with young people (e.g., art, music, text messaging, storytelling, poetry).

## Conclusions

Fewer studies than expected contributed to understanding the relationships between social connectedness and disconnectedness and suicidal experiences in people with recent onset psychosis. This was surprising because people with recent onset psychosis are most vulnerable to both social disconnection or detachment and suicidal experiences [11, 134]. The limited empirical evidence underscores that our understanding of the complex and multifaceted relationships between social connectedness and disconnectedness and suicidal experiences is insufficient. Furthermore, it highlights a potentially critical role of social disconnectedness in shaping suicidal experiences in individuals with recent onset psychosis. Social isolation, feeling thwarted and feeling like a burden to others significantly increase the risk of suicidal experiences [34, 58, 109, 111]. Addressing these social factors through targeted psychological interventions and meaningful supportive environments seems essential for effective suicide prevention in this vulnerable population. Notably, there is a lack of studies examining social connectedness within the expanding landscape of online social media forums and digital communication channels. This is a significant gap, as online environments may alleviate and intensify experiences of disconnectedness, including in individuals with recent onset psychosis. Without systematic investigation, our understanding of the impact of online social contexts on mental health, wellbeing, and suicidal experiences remains incomplete. Further research is needed to deepen our understanding and develop tailored strategies that consider the unique challenges faced by individuals with recent onset psychosis both in offline and online social contexts.

## Electronic supplementary material

Below is the link to the electronic supplementary material.

Supplementary Material 1

## Data Availability

No datasets were generated or analysed during the current study.
