# Supplementary material for: Social connectedness and disconnectedness in individuals with recent onset psychosis and suicidal experiences: a systematic review of the evidence
Source: BMC Psychiatry. 2026 May 13;26:524. doi: 10.1186/s12888-026-08092-z (PMC13347889; doi:10.1186/s12888-026-08092-z)
Supplement: Supplementary file 1 — Supplementary Material 1 [file 12888_2026_8092_MOESM1_ESM.docx]

**Supplementary Table S1**

*Search Terms Used in the Four Electronic Databases (i.e., Web of Science, PsycInfo, Embase, and MEDLINE)*

| **Category** | **Boolean Terms** |
| --- | --- |
| Recent onset psychosis | (FEP OR “first episode psychos*” OR “first-episode psychos*” OR “first episode psychot*” OR “first-episode psychot*” OR “first episode schizophrenia” OR “first-episode schizophrenia” OR “schizo*” OR UHR OR “ultra hight risk” OR “ultra-high risk” OR “clinically high risk” OR “clinically-high risk” OR “clinical high risk” OR CHR OR “at risk mental state*” OR “at-risk mental state*” OR “sub-clinical psycho*” OR “subclinical psycho*” OR “sub-threshold psycho*” OR “subthreshold psycho*” OR prodrome OR prodromal OR “early psychos*” OR “early psychot*” OR psychosis OR “psychot*” OR “recent onset psychos*” OR “recent-onset psychos*” OR “recent onset psychot*” OR “recent-onset psychot*” OR “paranoi*” OR “suspicious*” OR “delusion*” OR “persecutory delusion*” OR “psychosis early intervention*” OR “early intervention*”) AND |
| In-person interactions/  interpersonal relationships | (“social interact*” OR “social relation*” OR “social inter-relation*” OR “social interrelation*” OR interpersonal OR “social interconnection” OR “social connect*” OR “social standing” OR “social inclusion” OR “belong*” OR “social communit*” OR “communit*” OR “social communicat*” OR “communicat*” OR “social contact*” OR “social support*” OR “support group*” OR “peer group*” OR “peer support” OR “peer relation*” OR “friend* group*” OR “friend* circle*” OR “friend* relation*” OR “friend* network*” OR “family support” OR “family relation*” OR “intimate relation*” OR “romantic relation*” OR “emotional relation*” OR “partner relation*” “sexual encounter*” OR “sexual intimac*” OR “sexual relation*” OR “membership*” OR “network*” OR “social network*” OR “attachment style*” OR “attachment type*” OR “adult attachment” OR mattering OR “anti-mattering” OR loneliness OR lonely OR loner OR “alone*” OR “isolat*” OR “burden*” OR “social isolat*” OR “social stigma*” OR “stigma*” OR “self-stigma*” OR “social connect*” OR “social disconnect*” OR “social exclusion*” OR “exclusion*” OR “social reject*” OR “group reject*” OR outcast OR “mistrust*” OR “threat*” OR bully OR “bullie*” OR |
| Online interactions/  social media | “online network*” OR “online communicat*” OR “online platform*” OR “online social*” OR “online forum*” OR “online social activity” OR “online social network*” OR “online social media platform*” OR “social media” OR “social media activity” OR “social media network*” OR “social media platform*” OR “social media interact*” OR “social media website*” OR “social media site*” OR “social network* website*” OR “social network* site*” OR “smartphone app*” OR “mobile phone app*” OR ghosting OR gaming OR “gaming communit*” OR blog or “chat room*”) AND |
| Suicidal experiences | (suicid* OR “suicide attempt*” OR “suicid* plan*” OR “suicid* intent*” OR “suicid* behav*” OR “suicid* act*” OR “suicid* urge*” OR “suicid* compulsion*” OR “suicid* impuls*” OR “impulsive suicide” OR “suicid* image*” OR “image* of suicid*” OR “suicidal thought*” OR “suicid* idea*” OR “suicide method*” OR “suicide means” OR “death wish*” OR "passive suicid*" OR “self-annihilat*” OR “self annihilat*” OR “self-destruct*” OR “self destruct*” OR “self-injur*” OR “self injur*” OR “self-harm*” OR “self harm*” OR "death by suicide" OR “suicid* death*” OR "successful suicide" OR "completed suicide" OR "copycat suicide" OR “commit* suicid*”) |
| *‘NOT’* terms | NOT (euthanasia OR “assisted suicid*” OR “assisted dying” OR “assisted suicid*”OR NSSI OR “non-suicidal self-injury” OR “non-suicidal injury” OR “neuropsycholog*” OR “neuropsychopharmacolog*” OR “neuroimag*” OR “brain imag*” OR “pharmacolog*” OR “pharmac*” “medicine*” OR “medication*” OR “infant*” OR “pediatric*” OR “paediatric*” OR “pregnan*” OR antenatal OR prenatal OR “pre-natal” OR perinatal OR postnatal OR “post-natal” OR maternal OR “obstetric*” OR postpartum OR “post-partum” OR birth OR childbirth OR miscarriage OR stillbirth) |

**Supplementary Table S2**

*Quality Assessment Tool*

| **Study Design** | **Methodological Criteria** | **Responses** | | |
| --- | --- | --- | --- | --- |
|  |  | **Yes** | **No** | **Can’t tell** |
| *Screening questions for all study types* | S1. Are there clear research questions? |  |  |  |
|  | S2. Do the collected data allow the research questions to be addressed? |  |  |  |
| 1. Qualitative  *(e.g., grounded theory, ethnography, phenomenology, narrative research, case study, qualitative description)* | 1.1. Is the qualitative approach appropriate to answer the research question? |  |  |  |
|  | 1.2. Are the qualitative data collection methods adequate to address the research question? |  |  |  |
|  | 1.3. Are the findings adequately derived from the data? |  |  |  |
|  | 1.4. Is the interpretation of results sufficiently substantiated by data? |  |  |  |
|  | 1.5. Is there coherence between qualitative data sources, collection, analysis and interpretation? |  |  |  |
|  | 1.6. Has the relationship between researcher and participants been adequately considered? *(CASP item 6)* |  |  |  |
| 2. Quantitative non-randomised  *(e.g., prospective longitudinal, retrospective, case-control, cross-sectional analytic studies* | 2.1. Are the participants representative of the target population? |  |  |  |
|  | 2.2. Are measurements appropriate regarding the outcome or exposure? |  |  |  |
|  | 2.3. Are there complete outcome data? |  |  |  |
|  | 2.4. Are the confounders accounted for in the design and analysis? |  |  |  |
|  | 2.5. During the study period, did the exposure occurred as intended? |  |  |  |
| 3. Quantitative descriptive  *(e.g., survey studies, incidence/prevalence studies without a comparison group)* | 3.1. Is the sampling strategy relevant to address the research question? |  |  |  |
|  | 3.2. Is the sample representative of the target population? |  |  |  |
|  | 3.3. Are the measurements appropriate? |  |  |  |
|  | 3.4. Is the risk of nonresponse bias low? |  |  |  |
|  | 3.5. Is the statistical analysis appropriate to answer the research question? |  |  |  |
